# MicroRNA-146a: A Dominant, Negative Regulator of the Innate Immune Response

**DOI:** 10.3389/fimmu.2014.00578

**Published:** 2014-11-21

**Authors:** Reuben Saba, Debra L. Sorensen, Stephanie A. Booth

**Affiliations:** ^1^Molecular PathoBiology, National Microbiology Laboratory, Public Health Agency of Canada, Winnipeg, MB, Canada; ^2^Department of Medical Microbiology, University of Manitoba, Winnipeg, MB, Canada

**Keywords:** innate immunity, microRNAs, toll-like receptors, neurodegeneration

## Abstract

MicroRNAs (miRNAs) are a class of small non-coding RNA molecules that can play critical roles as regulators of numerous pathways and biological processes including the immune response. Emerging as one of the most important miRNAs to orchestrate immune and inflammatory signaling, often through its recognized target genes, IRAK1 and TRAF6, is microRNA-146a (miR-146a). MiR-146a is one, of a small number of miRNAs, whose expression is strongly induced following challenge of cells with bacterial endotoxin, and prolonged expression has been linked to immune tolerance, implying that it acts as a fine-tuning mechanism to prevent an overstimulation of the inflammatory response. In other cells, miR-146a has been shown to play a role in the control of the differentiation of megakaryocytic and monocytic lineages, adaptive immunity, and cancer. In this review, we discuss the central role prescribed to miR-146a in innate immunity. We particularly focus on the role played by miR-146a in the regulation and signaling mediated by one of the main pattern recognition receptors, toll/IL-1 receptors (TLRs). Additionally, we also discuss the role of miR-146a in several classes of autoimmune pathologies where this miRNA has been shown to be dysregulated, as well as its potential role in the pathobiology of neurodegenerative diseases.

## Introduction

Innate immunity is the critical first line of defense against invading microbial pathogens, and is the primary effector of the inflammatory response and initiator of the adaptive immune response. Microbial pathogens are recognized by several families of pattern recognition receptors (PRRs) that interact with conserved molecular structures that are broadly conserved on most microorganisms, which are known as pathogen associated molecular patterns (PAMPs) or danger associated molecular patterns (DAMPs) (1). These interactions trigger a cascade of intra-cellular signaling that results in the induction of transcription of pro-inflammatory cytokines and interferons (IFNs).

**Figure 1:**
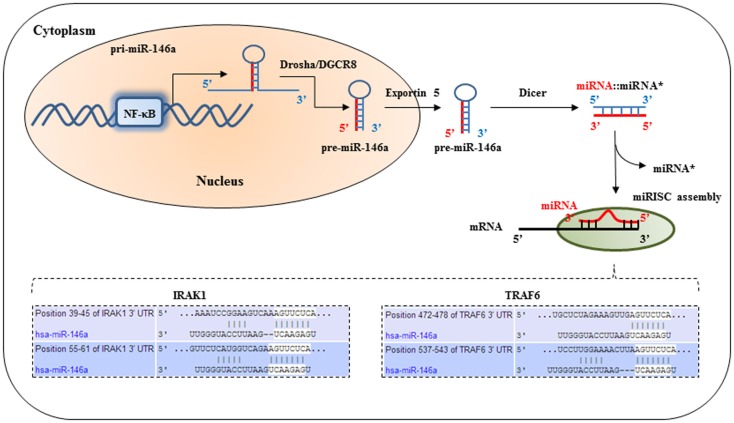
**IRAK1 and TRAF6 are molecular targets of miR-146a**. After the primary transcript of miRNA-146a is generated by RNA polymerase, the primary miRNA is processed by Drosha in the nucleus to produce a short hairpin pre-cursor miRNA (pre-miRNA) transcript, which is then shuttled into the cytoplasm. Further processing in the cytoplasm by Dicer produces the final mature 22-basepair dsRNA. This mature miRNA complex is then incorporated into the RNA-induced silencing complex in the cytoplasm. The seed-sequence binds to complimentary sequences in the 3′untranslated region (UTR) of its target mRNA transcripts, such as IRAK1, and TRAF6. This results in translational repression of IRAK1 and TRAF6.

The best characterized PRRs in regards to their microbial ligands, downstream signaling, and the effectors that are induced are undoubtedly members of the toll receptor family (2). Other families include the retinoic acid-inducible gene I (RIG-I)-like receptors and Nod-like receptors. Downstream signaling following stimulation of these receptors is intricately regulated by a huge variety of extra-cellular and intra-cellular mechanisms involving kinases, phosphatases, transcriptional co-activators, membrane proteins, and trafficking molecules, among others. Recently, a novel post-transcriptional mechanism mediated by small RNA species known as microRNAs (miRNAs) has been added to the list of important regulators of PRR signaling (Figure 1).

MiRNAs were first identified in 1993 by the laboratory of Victor Ambros (3). They found that a 22 nucleotide long RNA, transcribed from the lin-4 gene, could bind to the 3′ untranslated region (UTR) of the lin-14 messenger RNA (mRNA) of *C. elegans* through Watson and Crick complementary base-pairing and thereby inhibit the translation of the mRNA into a functional protein. At first thought to be an idiosyncrasy of this particular species, miRNAs were soon discovered to be present in a wide assortment of vertebrate species and cell types and it has become evident that these molecules exhibit important functions in the post-transcriptional regulation of protein synthesis (4).

Mature miRNAs are transcribed as pre-cursor transcripts either as independently or polycistronic clusters, or as introns of coding genes (5, 6). Their expression is generally highly regulated in tissue- and development-specific expression patterns, implying an important contribution to cell-type specific protein expression profiles. Due to their mode of function, miRNAs are especially well suited to be part of auto-regulatory feed-back loops. In particular cases, they are able to directly target their own regulators, thereby being able to “fine-tune” their own expression (7, 8). MiRNAs interact with their target genes by binding to complementary sequences in the 3′UTR of the target mRNA(s), see Figure 1. Most important for efficient binding is the continuous base-pairing of the “seed-region,” which is represented by miRNA nucleotides 2 → 8 in the 5′ region (9). In most animals, however, the overall base-pairing is imperfect leading to inhibited protein synthesis that is mediated by either translational repression and/or a promoting effect on mRNA deadenylation and decay.

The discovery of hundreds of experimentally verified miRNAs in mammals (http://www.mirbase.org/) has highlighted the importance of miRNA-dependent mechanisms as pervasive regulators of the genome. Since on average each miRNA is able to repress hundreds of target mRNAs (10, 11), computational analysis predicts that as much as 30–60% of mammalian genes may be controlled by these molecules (12). By targeting a plethora of mRNAs, miRNAs can regulate multiple pathways and biological processes simultaneously. Nevertheless, many miRNAs have now been found to not only participate in these large-scale processes but also to “fine-tune” the accurate levels of their target genes in a wide assortment of cells and tissues (13, 14). The first indication that miRNAs may play a role in the immune response originated from work that showed the selective expression of several miRNAs in cells of the immune system (15). Since then, numerous studies have revealed important physiological roles for miRNAs in the regulation of both the innate and adaptive immune response. In particular miR-146 and miR-155 are negative regulators of immune-related signaling pathways. MiR-155 and miR-21 have been shown to regulate immune tolerance and miR-223 and miR-150 play functional roles modulating the translation of transcription factors that are essential for the development of specific immune lineages. Moreover, their altered expression has been associated with pathological conditions of the immune system, including hematologic cancers and autoimmunity.

One miRNA that has been determined to play a central role in immune responses, particularly the innate immune response, is miR-146a. Its importance was recently confirmed in a miR-146a knock-out mouse model. In addition to developing autoimmunity, these mice are hyper-responsive to lipopolysaccharide (LPS) and demonstrate an exaggerated pro-inflammatory response when challenged with endotoxin. Furthermore, aged knock-out mice develop tumors in their secondary lymphoid organs and undergo myeloproliferation, suggesting that miR-146a regulates proliferation in immune cells (16, 17). Consistent with this latter finding, miR-146a-deficient mice develop myeloid malignancies due to chronic dysregulation of NF-κB signaling (17). Interestingly, miR-146a deficient mice develop many of the same abnormal hematopoietic phenotypes described in a subset of myelodysplastic syndrome (MDS) patients with 5q-syndrome in which a segment of chromosome 5q is deleted. This observation is quite interesting because miR-146a resides on chromosome 5q33.3 and because miR-145 and miR-146a expression is reportedly lost in the CD34^+^ bone marrow-derived progenitor cells of many MDS patients with 5q-syndrome (18). In addition, *in vitro* knockdown of miR-146a in mouse hematopoietic stem/progenitor cells can recapitulate many of the phenotypic abnormalities observed in 5q-syndrome. Taken together, these studies suggest a role for miR-146a as a tumor suppressor and in controlling the proliferation capabilities of immune cells. This involvement in cell growth and division likely leads to the high frequency with which alterations in expression is associated with malignancy.

Similar to miR-155, miR-146a expression is enriched in regulatory T (Treg) cells. Knock-out of miR-146a expression in the Treg cells of mice leads to a fatal breakdown of tolerance and results in a CD4^+^ helper T lymphocyte (Th1)-mediated immunopathology that is dependent on interferon gamma (IFN-γ). Furthermore, miR-146a’s targeting of the expression of Stat1, a member of the Signal Transducers and Activators of Transcription family of transcription factors that is involved in upregulating genes in response to interferon stimulation, is necessary for the *in vivo* suppressor function of Tregs (19). T effector subsets were also deficient for miR-146a in this model and were shown to produce higher levels of IFN-γ and to contribute to the pathogenic Th1 response. Further work utilizing lineage-specific ablation of conditional alleles is necessary to clearly define the role of miR-146a in different lymphocyte subsets.

In this review, we discuss the central role prescribed to miR-146a in innate immunity. We particularly focus on the role played by miR-146a in the regulation and signaling mediated by one of the main PRRs, TLRs. Additionally, we also discuss the role of miR-146a in several classes of autoimmune pathologies where this miRNA has been shown to be dysregulated.

## TLRs and miR-146a

Toll/IL-1 receptors are the best studied family of PRRs and were among the first pathways to be investigated for possible modulation by miRNAs. In human beings, there are 11 TLR homologs (TLR-1 to TLR-11), each specifically expressed with respect to cell type, and with unique extracellular or cellular domains that have the ability to recognize a wide variety of ligands (20). TLRs initiate the innate immune response by activating several signaling pathways that depend on adapter molecules, specifically, MyD88 (MyD88 signaling pathway) or the domain-containing adaptor protein inducing interferon-beta, TRIF, also known as TICAM1 (TRIF signaling pathway). Consequently, these adapter molecules mediate the downstream activation of the transcription factor nuclear factor kappa-light-chain-enhancer of activated B cells (NF-κB), mitogen activated protein kinases (MAPKs), members of the interferon regulatory factor family and activator protein-1 (AP-1), which contribute to the induction of pro-inflammatory cytokines, type 1 IFNs (IFNs such as IRF3 and IRF7) and anti-viral proteins (21, 22). The MyD88 pathway, which leads to the activation of NF-κB and MAPK, is used by most of the TLRs except for TLR3. TLR3 (and also TLR4) possess the ability to utilize the TRIF signaling pathway (23, 24).

Sufficient signaling by the TLRs is required for the effective clearance of pathogens but unrestrained TLR response can also be deleterious to the host. Therefore, TLR-mediated inflammatory response must be tightly controlled and the intricate regulatory potential afforded by miRNAs and miR-146a in particular is, therefore, highly pertinent to the physiological operation of this type of innate immune response. In this section, we discuss three particular scenarios by which miR-146a is intimately involved in the control of the mammalian innate immune response mediated by TLRs. This includes:

(i). The regulation of miR-146a expression by TLR-signaling.
(ii). The direct regulation of TLRs by miR-146a.
(iii). The regulation of TLR-signaling components/molecules by miR-146a.

### The regulation of miR-146a expression by TLR-signaling

**Figure 2:**
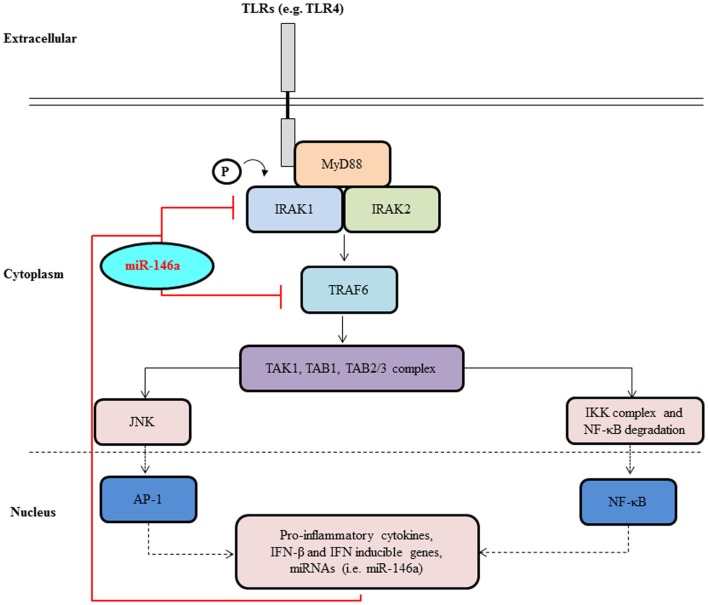
**MiR-146a negatively regulates signal transduction pathways leading to NF-κB activation**. Upon activation of a cell surface receptors, such as TLR4 belonging to the TLR family, a molecular cascade including TRAF6 and IRAK1 leads to IκBα phosphorylation and degradation and to NF-κB activation and nuclear translocation. NF-κB activation induces transcription of many genes, including pri-miR-146a. Once translocated to the cytoplasm and loaded onto the RISC complex, mature miR-146a contributes to the attenuation of receptor signaling through the down-regulation of IRAK1 and TRAF6.

The regulation of miRNA expression by TLR-signaling has been well documented with a wide assortment of miRNAs exhibiting altered expression. Nevertheless, a consensus has emerged that regardless of the TLR-stimulus utilized, the various screening platforms employed, or the cell types used, the induction of two miRNAs in particular, miR-146a and miR-155, are particularly prominent suggesting roles in the pathways downstream of TLR stimulation. The first set of evidence contributing to the role of miR-146a in innate immunity was acquired from work performed on the human monocytic THP-1 cell line in response to LPS stimulation or treatment with the pro-inflammatory cytokines, TNF-α, and interleukin-1 beta (IL-1β) (8). LPS, a TLR4 agonist, was found to rapidly induce the expression of miR-146a, which was then shown to act as a negative feed-back regulator of molecules in the same signaling system used for its own induction in an effort to dampen the magnitude of the immune response. MiR-146a has been shown both *in vitro* and *in vivo* to directly target two serine/threonine kinases, interleukin-1 receptor-associated kinase 1 (IRAK1) and tumor necrosis factor (TNF) receptor-associated factor 6 (TRAF6), that become associated with the interleukin-1 receptor (IL-1R) upon stimulation and are partially responsible for IL-1-induced upregulation of NF-kB. This binding results in the suppression of the expression of NF-κB’s target genes such as the interleukins IL-6, IL-8, IL-1β, and TNF alpha (TNF-α) (8, 16, 25–28) (Figure 2).

The induction of miR-146a by LPS, in response to TLR4-signaling, is dependent on NF-κB and the miR-146a promoter contains several putative binding sites (~3) for this transcription factor (8). Other TLR-agonists that have been shown to induce miR-146a expression, although to a lesser extent than LPS, include Pam3CSK4 (TLR2 agonist), peptidoglycan (TLR2 agonist), and flagellin (TLR5 agonist) (8, 29). Similarly, the infection of murine macrophages with vesicular stomatitis virus (VSV) induces miR-146a to negatively regulate the RIG-I anti-viral pathway by targeting not only IRAK2 and TRAF6, but also IRAK1, and in doing so suppressing the production of type-I interferon (i.e., IFNα) (27). MiR-146a has also been shown to target STAT-1 and IRF-5, both of which are involved in the type I interferon response pathway (28). Interestingly, low levels of miR-146a in lupus patients correlate with higher levels of interferon and with worse clinical manifestation (28). Taken together, the aforementioned studies strongly depict an extensive role for miR-146a in innate immunity as a dominant, negative regulator of the pro-inflammatory signaling cascade.

Studies have also prescribed a role for miR-146a in endotoxin-induced tolerance (26). Endotoxin tolerance is the hypo-responsive state of monocytes to subsequent challenge to LPS, following a period of prolonged LPS exposure. This particular tolerance is often necessary to prevent aberrant inflammation due to continuous exposure to bacterial components, such as those emanating from commensal microflora at epithelial surfaces. Experiments with THP-1 cells have shown that miR-146a levels increase following LPS exposure (TLR4-signaling) and negatively correlate with TNF-α levels as the cells develop a state of LPS tolerance (26). Importantly, tolerance induction requires miR-146a up-regulation and the transfection of exogenous miR-146a is sufficient to induce endotoxin tolerance, even in the absence of LPS-priming (26). Conversely, the knock-down of miR-146a diminishes the effects of LPS tolerance (29). Subsequent work confirmed these findings and showed that miR-146a was necessary for LPS-induced cross tolerance to several different TLR-ligands (29). Furthermore, miR-146a is important for innate immune tolerance in the neonatal intestine because it targets IRAK1 and thereby prevents apoptosis of intestinal epithelial cells following exposure to bacteria (30). As a whole, these studies support the key notion that although miR-146a is induced by TLR-signaling, its key role may be to serve as a dominant negative feed-back regulator to prevent uncontrolled inflammation in response to persistent bacterial exposures.

Two additional mechanisms of TLR4/LPS-signaling induced, miR-146a-mediated, endotoxin tolerance have been reported (31). Firstly, miR-146a was shown to be involved in the binding of the transcriptional repressor encoded by the RELB gene to the promoter region of TNF-α. The binding of RelB (NF-κB member) results in the generation of a facultative heterochromatin. The depletion of miR-146a alone is not sufficient to reverse the compact nature of the chromatin resulting in the persistence of endotoxin tolerance. It is only when both the RELB and miR-146a are depleted that the tolerance appears to be reversed. Secondly, miR-146a has also been shown to regulate the translational repression of TNFα. This is believed to be mediated through miR-146a preventing the interaction of the RNA-binding protein effector argonaute-2 (Ago2) and RNA-binding motif 4 (RBM4), both members of the translational repressor (miRNA/RISC) complex.

Consistent with the previous themes, the treatment of A549 lung epithelial cells (and also a range of other lung-associated cells including airway epithelial BEAS2B cells, primary human airway epithelial cells and primary human airway smooth muscle cells) with high levels of IL-1β rapidly increases miR-146a and suppresses the IL-1β-induced chemokines, IL-8 and RANTES (32, 33). Interestingly, in A549 lung cells, this negative feed-back inhibition was only apparent at high IL-1β concentrations suggesting a potential role for this miRNA-mediated mechanism during severe inflammation (32). The induction of miR-146a by IL-1β is mediated by both a mechanism involving NF-κB and also dependent on a JNK-1/2-mediated mechanism (33). More in depth analysis of the mechanism of miR-146a induction using pharmacological inhibitors revealed that although NF-κB is involved in the transcription of pri-miR-146a, the MAP kinases, ERK-1/2, and JNK-1/2 pathways appear to regulate the post-transcriptional processing of the mature miR-146a (34). Furthermore, miR-146a is constitutively expressed at high levels in non-activated Langerhans cells (LCs) in the epidermis upon comparison to interstitial dendritic cells in connective tissues (35). The transcription factor PU.1 induces miR-146a in response to TGF-β1 signals in the epidermis, and miR-146a increases the activation threshold for LCs in response to TLR2-dependent activation. These findings again support a role for miR-146a to negatively regulate the innate immune response in epithelial surfaces and suggest a mechanism for inducing an innate tolerance to commensal microflora.

Although the molecular mechanism(s) have yet to be fully elucidated, the induction of miR-146a by TLR7 and TLR9 agonists has now been shown in primary plasmacytoid dendritic cells (pDCs), where these receptors are clearly expressed (36). pDCs are one of the main sources of type 1 interferons during microbial infections and TLR7 and TLR9 sense the nucleic acids from these types of infections. Additionally, activated pDCs also generate pro-inflammatory cytokines and promote the up-regulation of co-stimulatory molecules, which in turn promote the priming of the adaptive immune response mediated by T-, B-, NK-, and dendritic-cells. The ectopic expression of miR-146a in the pDC cell line, CAL-1, impaired TLR-mediated signaling through reduction in the expression of IRAK1 and subsequently NF-κB activation. Moreover, the enforced expression of miR-146a in these cells also reduced the viability of the cells and also their role in the co-stimulation of the adaptive immune response. Interestingly, the induction of miR-146a by TLR7 and TLR9 activation appears to be a nature of the cell types used as primary cells (i.e., primary PMBCs or pDCs) appear to be more conducive for miR-146a induction than immortalized cell lines (i.e., THP).

Though a clear picture regarding the induction of miR-146a expression by various TLR-agonists appears to be emerging, whereby NF-κB appears to be intimately involved at the transcriptional level, less is known about the post-transcriptional stage and how the mature form of the miRNA is produced. Early evidence with a range of lung-associated cells suggests that MAP kinases may be involved. Another outstanding question in the field of miR-146a biology is whether there are TLR-signaling mechanisms that can decrease this and other host miRNA expression and whether this is related to disease pathobiology. Potentially, this could be through transcriptional repression and/or through post-transcriptional mechanisms that may destabilize pri- and pre-miRNA transcripts. At this conjuncture, however, it would be premature to dismiss that these topics are not being actively investigated. Alternatively, TLR-induced miR-146a induction may also be due to the accelerated processing of pre-miRNA transcripts and/or even through the delayed degradation of the mature miR-146a. However, at this time, evidence to support these claims is also very limited. Moreover, detailed investigations are also required to delineate the role of other miRNAs that appear to be regulated by TLR-signaling, alongside miR-146a, including but not limited to miR-155, miR-21, miR-132, and miR-146b, and also the time period after stimulation that these miRNAs are induced. Whether their role is to promote the pathogenesis of the infection, while miR-146a regulates the magnitude of the immune response, is yet to be resolved and would certainly provide a more compelling picture of the role of miRNAs in regulation of the vertebrate innate immune response. MiR-146b must also be mentioned here as it is very closely related to miR-146a, differing only by two nucleotides in its stem-loop region. It is located on a different chromosome to miR-146a but has an identical seed sequence and thus the same bioinformatically predicted targets. In fact, it has also been shown to negative regulate inflammation by interaction with IRAK1 and TRAF6 mRNAs. In contrast to miR-146a, however, it is expressed with delayed kinetics in respect to miR-146a in human monocytes in response to LPS stimulation and it has been shown to be induced by IL-10 via a STAT3-dependent loop (37). In a very recent study, miR-146b induction by STAT3 activation was shown to be epigenetically regulated, its expression being silenced in cancer cell lines and tumor tissue via methylation (38). In this situation, miR-146b appears to function as a tumor suppressor, thus highlighting how context-dependent expression and transcriptional regulation can provide miRNAs with identical seed sequences and putative targets with unique biological functions.

### The direct regulation of TLRs by miR-146a

To activate the immune response, pathogen recognition receptors (PRRs) must first recognize infecting pathogens. Thus, it might seem that the first potentially effective point at which to manipulate the TLR-signaling pathway is at the level of receptor expression (22, 39). However, to date, there are not many studies that can prove that TLRs themselves are directly targeted by miRNAs. It has been suggested that there are very few highly conserved target sites in the 3′-UTRs of TLR mRNAs (22). The miRNA target prediction program TargetScan showed that TLR coding genes have very few highly conserved target sites for miRNAs, and further analysis of the human 3′-UTR TLR region revealed that TLR genes are targeted by miRNAs mainly through weak, non-conserved sites (22, 39).

The mRNA encoding TLR4 has been shown to be regulated by members of the let-7 miRNA family. TLR4 mRNA contains the let-7e target site, and induction of let-7e expression in mouse macrophages decreases cell surface expression of this receptor (22, 40). The mRNA encoding TLR4 has also been shown to be targeted by other isoforms of the let-7 family. In human epithelial cells, TLR4 mRNA is regulated by let-7i (39, 41). Heikham and Shankar (42) using bioinformatic analysis to predict active miRNA sites in the 3′UTRs of TLRs showed that myeloid specific miR-23a was a strong candidate for regulating both TLR4 and TLR 3 expression.

Interestingly, more recently, Yang et al. (43) using a consensus bioinformatics approach, showed a possible interaction between TLR4 and miR-146a (39). With sustained oxidized low-density lipoprotein (oxLDL) stimulation, a decrease in expression of miR-146a appears concomitant with upregulation of TLR4 expression. Conversely, it was also shown that TLR4 can be down-regulated by over-expression of miR-146a. A luciferase reporter assay showed that miR-146a was shown to be able to bind directly to the TLR4 3′UTR target sequence, thus potentially regulating both oxLDL accumulation and down-regulating TLR4-dependant intra-cellular signaling pathways (43, 44). For the most part, these studies do not confirm a direct role for the regulation of TLR4 expression by miR-146a and, therefore, more detailed work is required. Although these data suggest that miR-146a has the potential to regulate at the level of TLR expression *in vitro*, much more experimental evidence exists to suggest that its action is most effective by regulating downstream signaling intermediates such as IRAK1 and TRAF6, transcription factors and cytokines, to specific modulation of various parts of the signaling cascade, rather than a non-specific knock-down of all TLR signaling as would be the result of regulation at the level of the receptor itself (39).

### The regulation of TLR-signaling components/molecules by miR-146a

In 2006, Taganov et al. (8) published the first report, high-lighting the vast potential for miRNA regulation of the innate immune response by analyzing the expression of 200 miRNAs after exposure to THP-1 cells to LPS. Through promoter studies, they determined that the induction of transcription of miR-146a by various microbial components and pro-inflammatory mediators such as LPS, TNFα, and IL-1β is critically dependent on NF-κB activation (8). IRAK1 and TRAF6 are key adapter molecules in TLR and IL-1 receptor signaling cascades mediating activation of NF-κB and AP-1 pathways. These proteins are important components of the myeloid differentiation primary response protein 88 (MyD88) dependent pathways for NF-κB activation downstream of TLR2, TLR4, TLR5, TLR7, TLR8, and TLR9 (8, 22). Multiple target sequence sites for miR-146 were found in the 3′UTRs of mRNAs encoding IRAK1, and TRAF6. Through luciferase reporter assays, it was shown that miR-146a can bind to multiple target sequences within the 3′UTR of IRAK1 and TRAF6 to regulate their expression. This groundbreaking research implicated miR-146 to be an important fine-tuner of the immune response by using negative feed-back regulation of TLR receptor and cytokine signaling (8). Once induced by NF-κB, miR-146a prevents the production of IRAK1 and TRAF6. However, proteins that have already been synthesized will continue to transduce signals, resulting in a built-in delay to the action of miR-146a leading to a gradual down-regulation of the inflammatory signaling cascade (45). Further studies have confirmed that miR-146a induction is indeed NF-κB dependent (17, 45).

In addition to targeting signaling proteins downstream of TLR receptors, studies have also shown that miR-146a decreases the expression of chemokines and cytokines, which are normally pro-inflammatory. These include suppression of CXC-chemokine ligand 8 (CXCL8) and CC-chemokine ligand 5 (CCL5) in epithelial cells (32), IL-6 and CXCL8 in fibroblasts and TNFα in the osteoarthritic tissue after IL-1 stimulation (46, 47), TNFα, IL-1β, and IL-6 by THP1 monocytes during LPS tolerance (26), type I IFNs by TLR7-stimulated peripheral blood mononuclear cells (PBMCs) and by Epstein-Barr virus (48, 49), and decreased expression of type I IFNs, TNFα, IL-1β, and IL-6 in macrophages during vesicular stomatis virus (VSV) infection or during LPS tolerance (22, 27, 39, 44). It is likely that most of these cytokines and chemokines are negatively regulated by targeted repression of IRAK1 and TRAF6 (22), and IRAK2 (27).

In 2008, Perry et al. examined miR-146a function by over-expression and inhibition and showed that IL-1β induced miR-146a expression negatively regulated the pro-inflammatory chemokines IL-8 and RANTES in alveolar A549 epithelial cells (32). To investigate the mechanism by which miR-146a might negatively regulate IL-8 and RANTES release, they first examined available databases to predict a number of potential targets. Not surprisingly, they found that IRAK1 and TRAF6 were prominent targets of miR-146a (30). Interestingly, they found that the expression and action of miR-146a was observed at a high IL-1β concentration, which indicated that this negative feed-back mechanism is only activated during severe inflammatory response (32). Further examination of the mechanism of action of miR-146a led them to believe that this response was unlikely to be mediated through the down-regulation of IRAK1 and TRAF6, and did not appear to act upon either IL-8 and RANTES transcription or secretion. Instead, in the case of IL-1β stimulation, they believed that miR-146a is involved in either direct or indirect targeting of IL-8 and RANTES translation (30, 32).

### miR-146a mediated regulation of RIG-1 like and NOD-like receptor signaling

RIG-1-like receptors (RLRs) constitute a family of three cytoplasmic RNA helicases that are critical for host anti-viral responses. They sense double-stranded RNA, a replication intermediate for RNA viruses, leading to the production of type I IFNs in infected cells. Many miRNAs can be induced by RIG-1 signaling, and can control viral replication through regulation of the RIG-I pathway and the expression of type I interferon (50). In 2009, Hou et al. first showed that in addition to two known miR-146a targets IRAK1 and TRAF6, that IRAK2, a kinase that compensates for IRAK1 to activate NF-κB, was a novel target of miR-146a (27, 39). Using TargetScan to predict new targets of miR-146a, they found that mouse IRAK2 had three putative miR-146a binding sites. To examine the possibility that IRAK2 was regulated post-transcriptionally by miR-146a, they cloned ~1 kb of the 3′UTR from mouse IRAK2 to the 3′UTR region of firefly luciferase gene or GFP gene and performed luciferase assays. They showed that transfection of miR-146a mimics decreased IRAK2 expression in macrophages at the protein and mRNA levels, whereas miR-146a inhibitors increased IRAK2 expression, suggesting that IRAK2 expression could be inhibited by miR-146a via both translational inhibition and mRNA degradation. As such, they reported that when up-regulated during viral infection, miR-146a is a negative regulator of the RIG1-dependant anti-viral pathway by targeting TRAF6, IRAK1, and IRAK2 (27). This group also further investigated the effect of miR-146a on VSV-induced production of pro-inflammatory cytokines and chemokines and found that miR-146a negatively regulated VSV-induced production of pro-inflammatory cytokines IL-1β, IL-6, and TNFα and chemokines, RANTES, in mouse primary macrophages (27).

The nucleotide-binding oligomerization domain (NOD) leucine-rich repeat-containing receptor (NLR) protein family coordinates intra-cellular surveillance to mediate innate immune responses and inflammation (51). There are several NOD leucine-rich repeat-containing receptor proteins and one of these, NOD2, acts as an important innate sensor that detects cytosolic PAMPs as well as DAMPs. NOD2 signaling activation in macrophages increases the expression of TNF-α, IL-6, cyclooxygenase 2 (COX-2), and iNOS, an enzyme, which produces nitric oxide that mediates bactericidal actions (52). It was recently reported that NOD2 signaling stimulated by muramyl dipeptide, a component of bacterial cell wall, up-regulates the expression miR-146a through transcription factors, NF-κB, PU.1, HSF2, and Oct-1 in mouse peritoneal macrophages (53). MiR-146a then directly targets the NUMB gene leading to its reduced expression and thereby relieves the suppression of the sonic hedgehog signaling pathway. Activation of this pathway ultimately leads to the expression of inflammatory mediators such as IL-12, TNF-α, IL-6, and the chemokines CCL-5, and CXCL-9. Although there are few publications to date that specifically describe a function for miR-146a in the regulation RIG-1 like and NOD-like receptor signaling many of its targets, such as the downstream adaptors IRAK1 and TRAF6, are shared. The pivotal roles of these targets in signaling pathways downstream of multiple PRR’s is highly suggestive of an overarching role for miR-146a in the activation of the innate immune response.

## An Emerging Role for miR-146a in Autoimmune Diseases and Disorders

The emerging role of miR-146a as a dominant negative regulator of the innate immune response has placed great emphasis on discovering a potential role for the miRNA in the etiology and/or pathobiology of a wide variety of autoimmune diseases and disorders. Although many miRNAs have been identified as being over-expressed and/or under-expressed in these types of pathologies, miR-146a in particular has been extensively reported by most investigators. Nevertheless, a harmonious consensus on the magnitude of miR-146a expression, or the lack thereof, in autoimmune pathological events has not yet been achieved. Several well defined autoimmune diseases and disorders where the aberrant expression of miR-146a has been identified include systemic lupus erythematosus (SLE), rheumatoid arthritis (RA), and Sjögren’s syndrome (SjS).

Systemic lupus erythematosus is a complex autoimmune pathology where tissue and organ damage are caused by the production of auto-antibodies, complement activation, and immune-complex deposition (54, 55). In the PBMCs of SLE patients, the expression of miR-146a was found to be down-regulated (28). The down-regulation negatively correlated with the severity of the disease and also with the interferon response. Elevated IFNα-levels in the serum of SLE patients is well known, along with the differential expression of IFN-inducible genes (56–59). Taken together, this “IFN-signature” has emerged as one of the most significant contributing factors in the pathobiology of SLE. Remarkably, the over-expression of miR-146a reduced, while the sequestration of endogenous miR-146a increased, the induction of type I interferons in PMBCs. Moreover, the miRNA directly repressed the trans-activation downstream of type 1 IFNs and can also directly target for post-transcriptional regulation IFN regulatory factor 5 and STAT-1. More importantly, miR-146a introduction into the PMBCs of affected individuals alleviated the IFN-signature associated with SLE. Genome wide association studies (GWAS) have also drawn attention to polymorphic regions in the genomic segments coding for miR-146a and SLE-susceptibility. Within the Asian population, the variant rs57095329 in the promoter region of the miR-146a gene, which is suspected of reducing efficient transcription factor binding (i.e., for Ets-1), contributes to the decreased expression of the miRNA and an increased risk to develop SLE (60). Similarly, an increased risk for SLE was identified in European individuals possessing the variant rs2431697 in the intergenic region of miR-146a, which reduces the expression of the miRNA (61). In contrast, screening for SLE-susceptible regions in the genomic segments around miR-146a did not show any significance in an alternate sampling of the Asian population (62). Taken together, these studies suggest that the under-expression of miR-146a in SLE may contribute to an aberrant IFN-signature during the course of the disease.

Rheumatoid arthritis is a phenotypically heterogeneous and chronically destructive inflammatory pathology of the synovial joints, often characterized by the presence of auto-antibodies in afflicted individuals (63). Evidence accumulated so far in the study of the disease, and specifically through the examination of the role of the miRNA in the disease, suggests that miR-146a is highly expressed in patients with RA (25, 64, 65). Increased expression of miR-146a in RA patients was observed in both the synoviam and PMBCs, and moreover, correlated with active disease. The hypothesis regarding increased expression of miR-146a and its potential role in RA stems from the findings that both IRAK1 and NF-κB are important molecules in octeoclastogenesis through the activation of osteoclast pre-cursor cells, as well as being direct and indirect targets of the miRNA, respectively (66, 67). Therefore, the increased expression of miR-146a and its subsequent down-regulation of IRAK1 and NF-κB may contribute to the repression of osteoclastogenesis and contribute to a decrease in joint destruction. In effect, the increased expression of this miRNA may serve as a putative biomarker of RA. Additionally, TNFα can also induce osteoclastogenesis and it has also been shown to lead to the expression of this miRNA. The over-expressed miR-146a seen here may be available to suppress osteoclastogenesis induced by TNFα. Linking polymorphisms in miR-146a genomic segments to RA susceptibility has been unsuccessful to date in various ethnic groups studied, including within the Greek and the Chinese populations (68, 69). In contrast, a polymorphism in the miR-146a 3′UTR binding site of IRAK1 has been shown to have some association with susceptibility to RA (69) and psoriatic arthritis (70).

Sjögren’s syndrome is a chronic autoimmune disease where focal lymphocyte infiltration and inflammation are observed in exocrine glands (71). The increased expression of miR-146a has been observed in PMBCs and salivary glands of both SjS-prone mice and SjS-patients (71, 72). In theory, the up-regulation of miR-146a should lead to a concomitant decrease in the expression of pro-inflammatory cytokines but in SjS-patients, the inverse has been shown to be true (73–76). This enigmatic finding may be attributed to some unknown mechanism that disables the ability of miR-146a to recognize its own set of target genes, leading to insufficient clearance of antigens and subsequent inflammation. One potential explanation for this ambiguous mechanism may be the differing expression patterns of miR-146a target genes IRAK1 and TRAF6 seen in SjS patients (72). With increasing miR-146a expression in PMBCs of SjS patients, a concomitant decrease in IRAK1 expression is observed whereas the expression pattern of TRAF6 increases. Potentially, the lack of a complete concerted down-regulation of NF-κB upstream molecules, in the presence of increased miR-146a expression, may account for this unique inflammatory response in SjS patients.

Taken together, the available evidence does indeed suggest a strong argument for the role of miR-146a in the pathobiology of autoimmune diseases and disorders. Nevertheless, more work is needed to clarify specific role(s) for the miRNA in the actual mechanism(s) of pathogenesis. Moreover, it should be noted that although a genetic component is strongly implicated in the etiolo-pathobiology of autoimmune diseases and disorders, the role of environmental factors should not be disregarded. The potential to use circulating miR-146a as diagnostic and/or prognostic biomarker of inflammation is a very strong possibility, as is the modulation of the expression of the miRNA through over-expression/knock-down approaches as a therapeutic counter strategy for inflammation induced by autoimmune diseases and disorders.

## An Emerging Role for miR-146a in Neurodegenerative Disorders

An intriguing and emerging area of research regarding miR-146a is its role in the central nervous system (CNS) where it has been shown to be robustly expressed, and where it may serve a pivotal role in regulating the unique innate immune response induced in these tissues in association with neurodegenerative disorders. Several studies have identified elevated expression of this miRNA in the brain and cerebrospinal fluid of patients with Alzheimer’s disease and age-related macular degeneration (77–79), in the brains of patients with multiple sclerosis (80), and in primary cultures of human neuronal and microglial cells stressed with IL-lβ, amyloid β, and inducers of oxidative stress (81–83). Additionally, in prion disease, a uniquely infectious neurodegenerative condition, up-regulation of miR-146a has been reported in the brains of human beings and mouse models of the disease (79, 84, 85). The expression of miR-146a has largely been studied in microglial cells of the brain, although miR-146a expression has also been noted in neuronal populations (85, 86). The expression of the miRNA in microglia cells is consistent with their role as the primary resident effector cells of the innate immune response in the brain, with many similarities to macrophages. Their activation is one of the first pathological features of a neurodegenerative condition (87). Activated microglia synthesize fairly low levels of pro-inflammatory cytokines, presumably as an inherent defense mechanism to prevent the severe pathology that can arise in host tissue as a result of an acute inflammatory response induced by rampant signaling in response to neurodegeneration. Nevertheless, microglia phenotypic changes are quite evident and are highly indicative of phagocytic capability.

**Figure 3:**
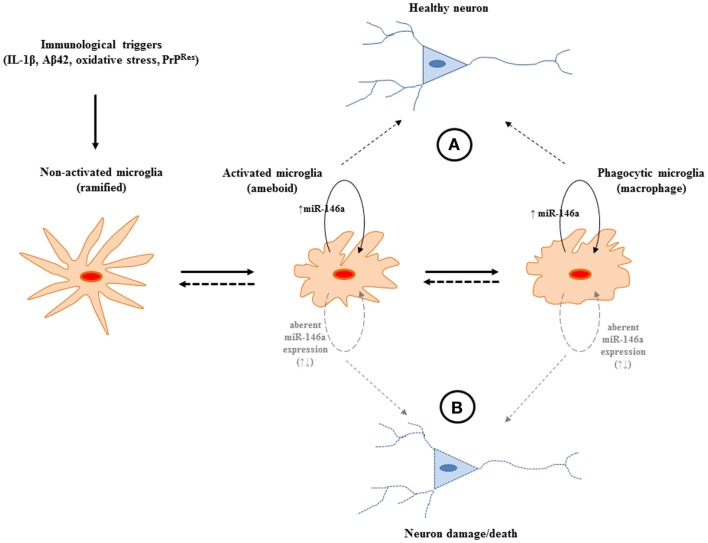
**A potential role for miR-146a expression in the central nervous system (CNS) during the course of neurodegeneration**. **(A)** The increased expression of the miRNA may provide protection from potentially harmful immune stimulation by regulating the activation of NF-κB. The miRNA may operate to keep the pro-inflammatory response of microglia “in-check” and may be beneficial during neurodegeneration. **(B)** Aberrant expression of miR-146a may lead to uncontrolled production of neurotoxic inflammatory factors (IL-1, TNFα, NO, NOO^−^, H_2_O_2_). Nevertheless, to define the exact sphere of influence of the miRNA, which has the potential to target multiple gene networks, more in depth studies are required.

Although miR-146a functionality has been extensively explored in macrophages, its role in microglia is just beginning to emerge and it may well have a pivotal role in keeping the microglial inflammatory response “in-check” so as not to exacerbate the neurodegenerative cascade that may ensue with an “un-checked” mechanism (Figure 3). Interestingly, the levels of miR-146a have also been shown to increase in aged microglia as well as macrophages and have been implicated in age-related dysfunction of macrophages (88, 89). Macrophages from aging mice exhibited a lack of response to stimulation with LPS and pro-inflammatory cytokines thus interrupting the negative feed-back regulation loop regulated by miR-146a. Aberrant NF-κB binding to the miR-146a promoter was shown to be associated with the abnormal expression of miR-146a in aged mice. High levels of histone deacetylase (HDACs) expression were also shown to contribute to the inhibition of miR-146a expression in LPS-stimulated macrophages from aged mice *in vitro*. Furthermore, a number of polymorphisms, have been identified that either interrupt its binding site in the 3′UTR region target genes IRAK1 and TRAF6, or affect the expression of miR-146a (68–70). These may have an association with age-related diseases such as RA (as previously mentioned) as well as Alzheimer’s disease (68, 90). Although metagenomic studies on miR-146a associated SNPs to date are limited by the small populations investigated, this is a promising strategy to determine prognostic and diagnostic markers that are focused on the non-coding and regulatory segments of the human genome. It is possible that these dysfunctions may be linked to the increased risk for disease of aging that have an inflammatory component such as neurodegenerative diseases and that miR-146a may be a target for treating such age-related disorders.

In order to fully elucidate the role of miR-146a in microglia cells and its role in neurodegeneration as a whole, identification of functionally important miR-146a target genes is warranted. In general, the inflammatory response to infections involves the induction of many genes, a process that must be tightly controlled not only to achieve the effective clearance of the infection but also to avoid consequences of dysregulated gene expression, such as uncontrolled inflammation. As only a handful of miRNAs have so far been linked to the molecular mechanisms of the CNS, and since neurodegenerative diseases present themselves with an atypical immune response characterized by the production of both pro- and anti-inflammatory mediators (91), studies are essential in understanding the specific component(s) of cellular immune response or other pathway(s) (i.e., morphological pathways that accompany activation, chemotaxis, and oxidative-burst) that may be regulated by miR-146a induction. Considering that no therapy is currently available for many human neurodegenerative conditions and since miRNAs simultaneously target numerous genes for silencing, investigations of this type may be beneficial for identifying potential new targets for therapeutic and/or compassionate intervention.

## Conclusion

Numerous studies have established a critical role for miR-146a in inflammation and immunity where it primarily appears to operate as a dominant negative regulator of the vertebrate innate immune response. This miRNA appears to “fine-tune” or “dampen” the innate immune response to possibly prevent an uncontrolled inflammatory response that may lead to the exacerbation of the initial infection. MiR-146a mainly operates in this manner through the targeting for down-regulation NF-κB upstream molecules IRAK1 and TRAF6, of the MyD88-dependent signaling pathway, leading to a concerted decrease in the production of various inflammatory mediators. Despite numerous studies that show the induction of miR-146a by pro-inflammatory mediators most notably of the TLR-signaling mechanism, there is still incomplete evidence regarding its precise role, mechanism of action, and the potential landscape of target genes regulated. Also relatively understudied is whether miR-146a is similarly induced by other vertebrate PRRs involved in innate immune signaling such as the signaling mediated by RIG-I-like receptors, AIM2-like receptors and Nod-like receptors, and also whether miR-146a serves a similar purpose in these pathways as it does in TLR-signaling. Although most studies on miR-146a function have been focused on investigating the TLR pathway and cells involved in inflammation, it is undoubtedly expressed in numerous other cell types and here may be involved in a further range of context-specific functions. Usage of the miR-146a^−/−^ transgenic mouse (16, 17) will aid in unraveling these, as will investigating the role of this miRNA in the pathogenesis of human diseases. It will then be an important next step to determine whether manipulating the levels of expression of miR-146a, as well as its targets, has therapeutic benefits.

## Conflict of Interest Statement

The authors declare that the research was conducted in the absence of any commercial or financial relationships that could be construed as a potential conflict of interest.
